# Self-Representations of Military Veterans Suffering From Chronic Post-traumatic Stress Disorder: The Role of Sport

**DOI:** 10.3389/fpsyt.2021.766515

**Published:** 2021-11-05

**Authors:** Celia Belrose, Anais Duffaud, Dominique Levy, Aida Beji, Sandrine Jacob, Gregory Lorion, Charles Martin-Krumm, Marion Trousselard

**Affiliations:** ^1^French Armed Forces Biomedical Research Institute, Brétigny-sur-Orge, France; ^2^Réseau ABC des Psychotraumas, Montpellier, France; ^3^Université Côte d'Azur, Nice, France; ^4^APEMAC/EPSAM, EA 4360, Metz, France; ^5^French Military Health Service Academy, Paris, France; ^6^Laboratoire VCR, École de Psychologues Praticiens de Institut Catholique de Paris (Catholic Institute of Paris), EA 7403, Paris, France

**Keywords:** post-traumatic stress disorder, veterans, body representation, person representation, sport, mind-body connection

## Abstract

**Background:** Post-traumatic stress disorder (PTSD) is a psychiatric illness that is very prevalent in both civilian and military environments. The clinical course, regardless of management, is chronic for a number of patients, especially veterans. Persistent PTSD symptoms interact with representations of the person and their body, and may negatively impact rehabilitation. Sport is known to help psychiatric patients such as those suffering from PTSD, as it improves the connection with the body, and supports physiological and emotional regulation. However, the impact of sport on self-representations has not yet been studied. The first aim of this study is to explore person and body representations in a population of military veterans suffering from chronic PTSD, as a function of clinical severity. Second, it aims to explore how a 9-day sport program, which includes an element of socio-professional rehabilitation, changes representations of the person and their body.

**Methods:** This exploratory qualitative study examined the self-representation of veterans with chronic PTSD before a sport rehabilitation program. Veterans were given the prompts “body” and “person” and asked to free associate. PTSD severity and the mind–body connection were assessed using the Posttraumatic Stress Disorder Checklist for DSM-5, and the Freiburg Mindfulness Inventory, respectively. Parasympathetic activity was recorded at rest. A subgroup of the population volunteered to participate in a post-program session to record the same semantic, psychological, and physiological variables.

**Results:** Although before the program, veterans gave more negatively than positively valenced words, no relation was observed between the overall number of negative words and PTSD severity. Post-program, changes were observed in terms of valence. Specifically, some negatively-valenced categories of words disappeared, and some positive categories appeared. At the same time, there was a fall in PTSD severity, an increase in the mind–body connection, and a decrease in parasympathetic activation.

**Conclusions:** This study highlighted that veterans with chronic PTSD have a negative representation of the self. A dedicated, 9-day program that included regular sport improved self-representations related to both the person and their body, and reduced PTSD symptoms. The findings underline the importance of ensuring that programs for patients suffering from chronic PTSD should include sporting activity, and highlight the benefits. Sport appears to be a path to the reappropriation of a positive image of the self, by improving the representation of the body. This relationship could be consistent with improved interoception, but our results need further investigation.

## Introduction

Post-traumatic stress disorder (PTSD) is an adaptive condition that can occur following one or more traumatic events that involve direct or indirect confrontation with death, threatened death, actual or threatened serious injury, or actual or threatened sexual violence (1). According to DSM-5, patients with PTSD present four clusters of symptoms: reliving, avoidance, hyper-reactivity, and negative cognitions and affects (1). Moreover, there can be numerous comorbidities (2). In addition to their clinical and cognitive symptoms, PTSD patients can suffer from the changes in the perception of self (3), leading to the sense of lost identity (4–6) that further degrade their quality of life, and impair their ability to maintain social and professional relationships.

In the general population, the lifetime prevalence of PTSD ranges from 1 to 7%, depending on the sample (7) and, for some patients, the condition becomes chronic (8). While studies show that more than half of patients make positive progress (9), a relatively recent review highlights that clinical outcomes can be highly variable (10): in particular, only 18–50% achieve stable remission. These figures are similar for military veterans (11–14), although the prevalence is estimated to be around 20% in this population, depending on the nature of the mission (15, 16). Studies also point out that the disorder can take many different paths (13, 14). Overall, once established, PTSD is a complex clinical entity that evolves in a variety of ways, becoming chronic in many patients.

One of the many impacts of PTSD is to destabilize the individual's identity. Notably, it can “impact the resources a person brings to identity work” (17). Studies in military cohorts have consistently found an association between identity and poor mental health (18), and between military identity and poor psychological functioning (19). Identity disruption, understood as a loss of temporal integration following a trauma, has been associated with reintegration problems in veterans suffering from poor mental health (20) which is consistent with an interrelation between identity and social validation (21). Social identity resources have been described as “the social psychological capacities that flow from internalized group memberships such as support, solidarity and belonging” [(6); p. 312]. Although it is clear that each of the two constructs impacts the other, the intersection between PTSD and identity remains poorly understood.

One reason for this may be the lack of clarity regarding concepts related to identity. The idea of identity is closely related to that of appraisal or beliefs about the self and the world. Although identity and the self are closely-related concepts, identity is considered to consist of representations and feelings, while the self is considered as a purely cognitive concept, the “self-concept” (22), which is, first and foremost, situated within the body. Furthermore, the self-concept refers to a representation of the self that encompasses the ways in which people see themselves, while self-presentation focuses on the way in which people present themselves to others (21).

Whether it be the self-concept or the self-presentation, both are based on the self-referential processing (SRP). SRP is an embedded process that consists of relating information to the self (23, 24), which is also considered as the “experiential self.” Frewen (25) considers that verbal SRP is a form of introspection that refers to the semantic self as the subject, while non-verbal SRP is a form of interoception that refers to the somatic self-object. Although both imply top-down and bottom-up controls, top-down control is tested using focused attention and executive tasks, while bottom-up control is mainly tested in a resting state using mind-wandering (for verbal SRP) and body-wandering (for non-verbal SRP) exercises.

To the best of our knowledge, little is known about the physiological correlates of SRP. At the same time, the physiological correlates of interoception are beginning to be described: the quality of interoception has been shown to be partly related to heart rate variability (HRV), notably high vagal tone (26–28). HRV has proven to be a reliable, non-invasive descriptor of the function of the autonomic/central nervous system, and is thought to reflect higher brain activities. In the case of PTSD, abnormalities associated with SRP have been clearly highlighted. Clinical symptoms are significant, as they are consistent with the negative self-referential and other-referential cognitive appraisals that a patient with PTSD suffers from. They include persistent and exaggerated negative expectations of oneself, as well as a distorted sense of self- or other-focused blame regarding the causes and consequences of the traumatic event (1). A patient with PTSD often experiences pervasive, negative emotional states, of which the most common are self-conscious, internally-directed emotions such as guilt and shame (29, 30). Symptoms related to SRP abnormalities may play an important role in the maintenance or exacerbation of the symptoms of PTSD, and the development of comorbidities. Consequently, their identification during PTSD treatment is critical although, to the best of our knowledge, there is no consensus regarding an experimental task that could measure verbal and non-verbal SRP (25). The above observations suggest that notions of the self could provide a useful theoretical framework to study PTSD; such an approach would both deepen our understanding and enhance care.

In this context, a recent review (31) highlights convergent results which suggest that sport practice combined with other forms of treatment may reduce symptoms above and beyond standard treatments alone. Although data evaluating the benefits of sport practice on identity disruption and/or self are scarce, some exploratory results seem relevant. In a single case study of a war and torture survivor, practicing sport was found to not only have beneficial cognitive and emotional effects, but also enhance the sense of belonging. Other positive impacts were a better sense of body and self, and an improved ability to cope with pain (32). In their review of the impact of sport practice on the well-being of combat veterans suffering from PTSD, Caddick and Smith (33) noted that the evidence underlines the value of sport practice in well-being and rehabilitation. Among veterans, regular practice of elite sport has been found to support a reconnection with their previous identity and sense of self. In the military context, developing a sense of belonging with peers is highly valued, and may contribute to bringing personnel back to good health.

Finally, studies consistently report reduced HRV in people with PTSD. Such findings suggest autonomic inflexibility due to sympathetic overactivity and/or parasympathetic insufficiency (34, 35). These results once again raise the question of the benefits of sport practice on HRV regulation for patients with PTSD. It is well-known that athletic condition is an important variable that influences the autonomic control of the heart (36). It has also been shown that endurance sports increase parasympathetic modulation over a 24-h period (37). Although some data suggest the value of sport in supporting parasympathetic improvement, there is currently no data that demonstrates the benefits with respect to PTSD symptoms.

In response to the lack of knowledge about self in the context of PTSD, and the lack of validated interventions that aim to improve the self-image, this study is the first to evaluate the verbal valence and semantics that veterans use to talk about themselves and their bodies, as a function of PTSD severity. First, we hypothesize that we will observe a correlation between the number of negative words used in verbal responses related to both the person and his or her body, and the clinical severity of PTSD symptoms. Second, we evaluate the therapeutic effects and psychophysiological processes observed during a sport's program organized by the French Army for veterans with PTSD. Here, we hypothesize that practicing sport decreases the severity of PTSD symptoms, through processes that improve the person's self-representation, and an improvement in the psychophysiological mind–body connection.

## Materials and Methods

### Participants

The population included in this exploratory study consisted of 47 veterans who had been on sick leave due to PTSD for over 6 months, between May 2018 and April 2021. This group had been enrolled on a professional reinsertion course following a recommendation regarding their suitability from their referring psychiatrist. These courses are organized by the French Army's wounded soldiers' unit (the *Cellule d'aide aux blessés de l'armée de Terre*). Five to six courses take place each year, with 8–12 participants. In addition to medical care, the aim is to support the recovery of psychologically injured soldiers, in order to facilitate their reintegration into civilian life. The program runs over a 9-day period, and is composed of group and individual physical activities, and professional exercises.

The study received prior approval from the Sud-Mediterranée III Personal Protection Committee (10 September 2018; NTC03995992). All subjects received information on the protocol, and gave written consent prior to participation.

### Procedure

For the full cohort of 47 veterans, baseline semantic, psychological and physiological data were collected before the program began. A subgroup of 26 individuals volunteered to participate in an experimental session at the end of the program. For this group, semantic, psychological and physiological data were evaluated twice (pre- and post-program).

The program takes place either in a small village in the Écrins National Park in France, for multisport activities such as hiking, mountain biking, climbing, canyoning, and orienteering, or in the *Domaine les Gueules Cassées* (a recreational site located near the Mediterranean Sea, initially dedicated to the care of wounded soldiers), for water sports. The daily program consists of around 3 h of sporting activities, and one or two practical, individual and group workshops focused on PTSD information, or coaching in professional skills.

Whatever the multisport program or the water sport program, each sport activity is supervised by a qualified sports instructor and begins with an explanation of the session and a warm-up and ends with a debriefing on the session. The main goal of each session is to get in touch with the sensations of the body without the idea of performance. Concerning the multisport program, the first day is a 2-h walk of about 6 km to get back into shape. If the conditions are favorable, the schedule for the classic program is as follows. The veterans have a climbing session on the second and fourth days. The level of difficulty of the climbing routes is progressive from 4 to 5+ according to the International Union of Mountaineering Associations. For days 3 and 5, the activity is a mountain bike activity in the mountains. The last 3 days are organized with a canyoning activity lasting all the day, and according to the preferences of the veterans of the climbing or the mountain bike activity. The last day is reserved for the orientation race of about 3 h with a difference in altitude of 400 m. The water sport program combines the discovery of swimming with fins and scuba diving. In order to reduce, or even eliminate the anxiety that is described with diving because of the environment, and the use of complex equipment, a very progressive training program was implemented with the recommendations of the Divers Alert Network. The protocol did not include any long dives at depths >20 m. The first two dives take place in a swimming pool. This make it easy to evaluate the level of anxiety of veterans, and to form homogeneous groups for dives in the sea. Throughout the course, the depths reached increased very gradually, and the ratio of veterans to monitors varies from two to one to one to one (for the most stressed individuals).

Professional activities focused on preparing a curriculum vita, coaching for professional resources and some role-playing for team building. During their free time, participants can join in group activities such as table soccer, pool, or other games, or simply relax. Civilian or military experts in human resources and social reinsertion conduct the workshops. A military psychologist is present at all times to provide support for symptoms of anxiety or substance dependence if one veteran asks for an emotional support during the 9-day program. There is no systematic psychotherapeutic treatment. All participants completed the full program.

### Variables

Sociodemographic information included age, gender, social environment, and total number of major stressors encountered in either the professional or the personal environment.

Two self-report questionnaires were used. The PTSD Check List Scale (PCL5) (1, 38) was selected to assess PTSD severity. The patient is asked to rate the following four categories of symptoms: re-experiencing; avoiding situations that remind the person of the event; hyperarousal; and impairment to cognitive and emotional functioning. Each self-descriptive statement is evaluated using a 4-point Likert scale ranging from (2) “not at all” to (4) “extremely.” Valid PCL-5 scores range 0–80 with higher scores indicating worse PTSD severity. Among the different thresholds that are proposed in the literature to diagnose PTSD, we chose the highest, as our aim was to identify a very specific group (1). Specifically, the cut-off point proposed by the National Center for PTSD—33—was selected.

The Freiburg Mindfulness Inventory-14 (FMI) was selected to assess the mind–body connection, as mindfulness has been consistently associated with higher interoceptive awareness (39). The FMI is a short form (14 item) self-report questionnaire that has been developed for people with no background knowledge of mindfulness (40, 41). It is considered to be a consistent and reliable scale for evaluating mindfulness, and is divided into two subfactors (42). “Acceptance” is the ability to embrace unwanted thoughts and “Presence” reflects a willingness to be present, characterized by a non-judgmental attitude to events that occur in the environment. Each self-descriptive statement is evaluated using a 4-point Likert scale ranging from (1) “strongly disagree” to (4) “strongly agree.” Valid FMI scores range 14–56 with higher scores indicating high mindfulness disposition.

The free association method was used to record verbal representations reported by the individual about themselves and their physical body. This method was initially developed in the field of psychoanalysis, and has more recently been adopted by cognitive and social psychology (43). It is used to collect the content of the participant's representational universe. Participants were given two prompts (describe yourself; describe your body) and asked to respond with up to 10 words or expressions that spontaneously came to mind.

HRV was assessed during a 5-min period at rest by electrocardiogram (ECG) signals captured using the PHYSIONER system (CODESNA, France). This clinical and research device uses a 250 Hz sampling rate to accurately detect R-wave peaks. The R–R interval corresponds to the beat-to-beat interval of the instantaneous heart rate, and temporal, frequential or non-linear analyses can be used to extract HRV information. For clarity, we chose to use only one feature: RMSSD (the root mean square of successive differences between normal R–R intervals). The benefits of using RMSSD include: (1) its resistance to the influence of breathing frequency, which is a problem in frequential measures (44); and (2) its ability to capture levels of parasympathetic activity over a short period. A RMSSD score for each subject was calculated using KUBIOS HRV analysis software. The ECG was always performed between 14:00 and 18:00 to limit variation in circadian rhythm.

### Statistical Analyses

Statistical analyses were performed using Statistica software (Statsoft France, Maisons-Alfort, version 7.1). Data are expressed as a number and percentage (%) for qualitative variables, and as the mean (M) and standard deviation (SD) for quantitative variables. The reliability of each psychological measurement (self-administered PCL5 and FMI questionnaires) was gauged by computing Cronbach's alpha (45). All were above 0.74, which indicates good reliability.

In the first step, we used the PCL5 median to categorize veterans into two groups: one where scores were above the median (the Severe PTSD Group), and another where scores were below the median (the Moderate PTSD Group). This qualitative inductive analysis used a manifest analysis with description of what the informants actually say with the objective to stay very close to the text, to use the words themselves, and to describe the visible and obvious in the text (46). First, words used to describe the person and their body were categorized into positive, negative and neutral valence, and counted. Second, the categorizing strategy was based on similarity-based relations that involve resemblances or common features. These two steps of valence coding as of categorization were carried out by three independent judges, who compared their own evaluations and reached a consensus.

Between-group comparisons of sociodemographic, psychophysiological, and semantic data were performed using Pearson's chi-square test for variables with several modalities, and either Student's *t*-test, or the non-parametric Kruskal–Wallis test for quantitative data.

The second step concerned subjects who had volunteered for the post-program session. Here, the aim was to evaluate the effects of the program on the words used to describe themselves and their body, with respect to their valence and semantic characteristics, PTSD symptoms, FMI scores, and RMSSD. A separate Wilcoxon non-parametric analysis was run, given the non-homogeneity of variance (Levene's test).

In each step, Bravais-Pearson correlation analyses were used. The statistical threshold for significance was set at *p* < 0.05. A trend was considered to be present when *p* < 0.10.

## Results

### Baseline Description of the Population

Four individuals were excluded from the analysis as they did not complete the questionnaires, leaving a total of 43 patients who had been suffering from PTSD for more than 6 months. The average duration of sick leave was 12 months (SD: 9.4). All of the population was male. Their average age was 33.95 years (SD: 6.02). Of these, 29 (67.44%) were living with a partner, eight (18.6%) were single, and six (13.95%) were divorced. Fourteen (35%) had at least one child. Mean number of major stressors encountered in either the professional or the personal environment was 3.3 (SD: 1.54) and 2.15 (SD: 1.49) respectively.

All subjects had a PCL5 score equal to, or above 33. Mean clinical severity (PCL5) was 61.42 (SD: 12.36). No correlation was found between the PCL5 score and the overall FMI score or its sub-scores, or between the PCL5 score and RMSSD.

The median PCL5 score for the group was 61. Twenty-one subjects were classified as suffering from moderate PTSD. The mean score within this group was 50.9 (SD: 5.6). Twenty-two participants were classified as suffering from severe PTSD, including one subject with a PCL5 score equal to the median. Here, the mean was 71.45 (SD: 7.71). There was no difference between the two groups in terms of age or duration of sick leave. RMSSD tended to be higher for the moderate group compared to the severe group, while no difference was observed for the overall FMI score, or its sub-scores, between the two groups (Table 1).

**Table 1:** Psychophysiological characteristics of moderate and severe PTSD groups.

	**Moderate PTSD (M; SD)**	**Severe PTSD (M; SD)**	***t* or H (*p*)**
FMI	32.20 (6.64)	30.87 (6.65)	*t*:0.63 (0.53)
FMI-P	14.90 (3.49)	14.52 (2.77)	*t*:0.38 (0.7)
FMI-A	17.30 (3.84)	16.35 (4.4)	*t*:0.73 (0.47)
RMSSD	0.1 (0.05)	0.08 (0.06)	H: 2.57 (0.1)

*FMI, Freiburg Mindfulness Inventory; FMI-P, FMI-presence; FMI-A, FMI-acceptance; M, mean; SD, standard deviation*.

### Baseline Free Association Results

As shown in Table 2, a total of 230 words were given by the 43 participants. Of these, 142 terms (61.7%) referred to the person, and 88 (38.3%) to their physical body. Of the 230 words, none were neutral, 76 (33.5%) were positive, and the remainder (66.5%) were negative. Overall, 36.95% of words were negative and referred to the person; 29.56% were negative words about the body; 24.78% were positive words describing the person; and 8.69% were positive words about the body. The distribution of words used to describe the person and their body differed between severe and moderate PTSD groups (*X*^2^ = 6.64, *p* = 0.01).

**Table 2:** Examples of positive and negative words for each category given by participants to describe their person and their body.

**Category**	**Positive**	**Negative**
**Person**		
Cognitive	*Perfectionist*	*Pessimistic*
Behavioral	*Athletic*	*Impulsive*
Affective	*Happy*	*Sad*
Volitional	*Competitor*	*No desire*
Moral	*Loyal*	*Evil*
Relational	*Smiling*	*Unbearable*
Identity	*Rather educated*	*Ambivalent*
**Body**		
Evaluation	*Protective*	*Disgust*
Vitality	*My body is alive*	*My body is sluggish*
Relaxed/tense		*Stressed*
Dys/functional	*My body is a tool*	*Damaged*
Physical image	*Thin*	*Overweight*
Pain		*Painful*

**Figure 1:**
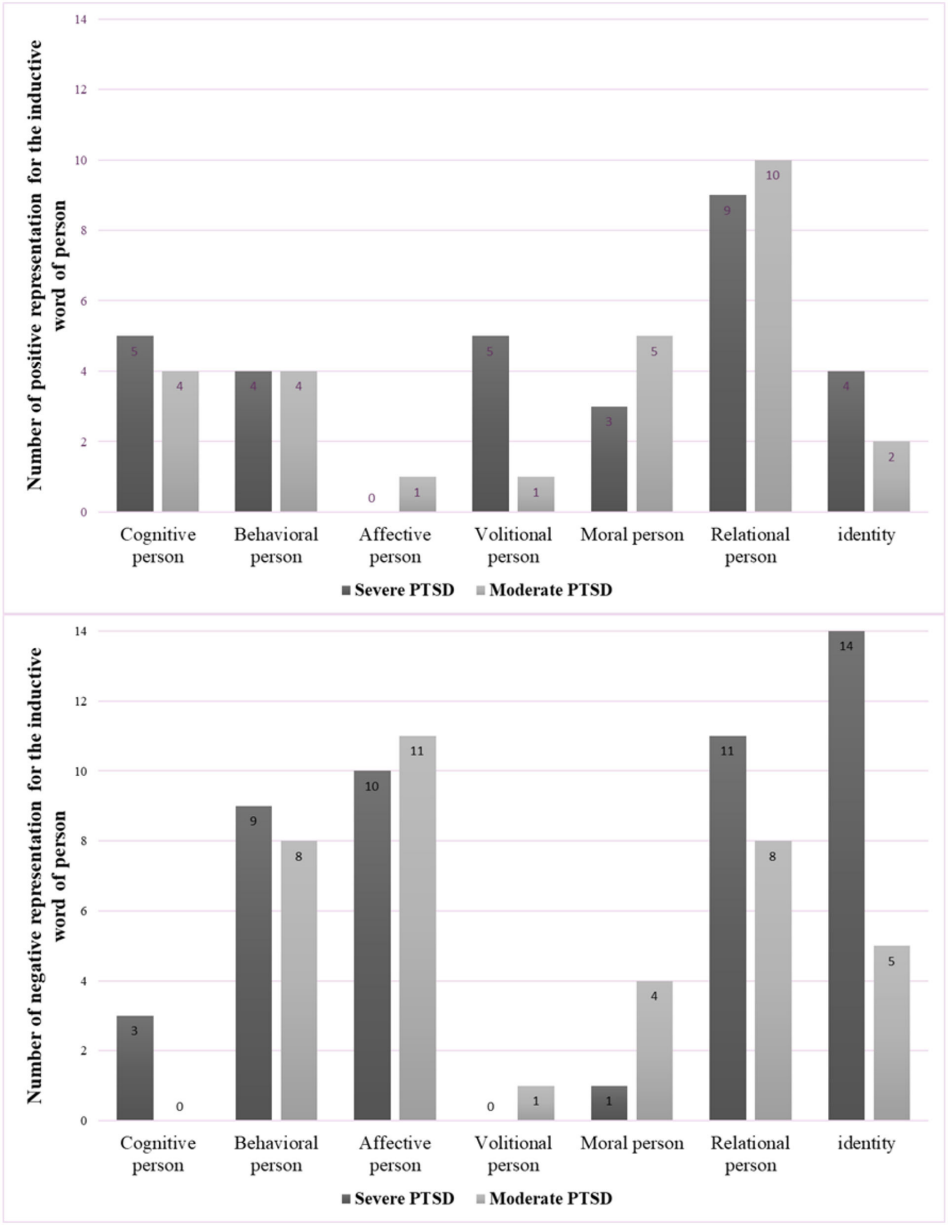
Categories and numbers of positive (top) and negative (bottom) words referring to the person for severe and moderate PTSD groups.

Regarding the 142 words associated with the person, 39% were positive and 61% were negative. Patients in the severe group gave 78 words in reference to themselves, 37% of which were positive and 63% negative. Patients in the moderate group gave 64 words, 42% of which were positive and 58% were negative. No difference between groups was observed, either in terms of the total number of words, or in terms of the number of positive and negative words. Seven overall categories were identified, which were split into subcategories according to their positive or negative valence. These seven categories were: cognitive, behavioral, affective, volitional, moral, relational and identity (Figure 1).

**Figure 2:**
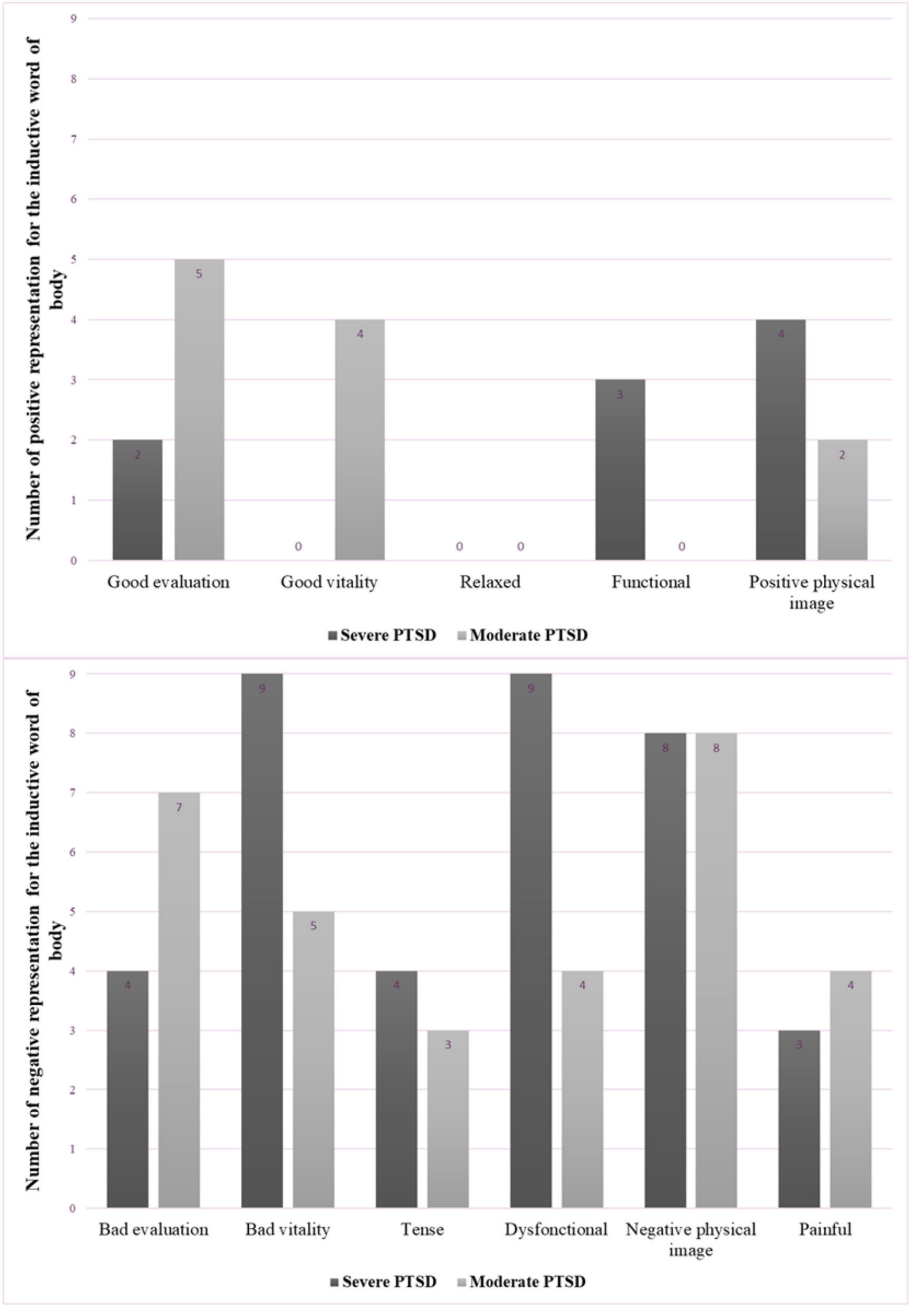
Categories and numbers of positive (top) and negative (bottom) words referring to the physical body, for severe and moderate PTSD groups.

Regarding the 88 words associated with the body, 23% were positive and 77% were negative. Patients in the severe group gave 46 words in reference to their own body, 20% of which were positive and 80% were negative. Patients in the moderate group gave 42 words, 26% of which were positive and 74% were negative. In this case, the distribution of valences differed between groups (*X*^2^ = 3.96, *p* = 0.04). Specifically, 42.05% of negative words were given by members of the severe group, compared to 35.22% for the moderate group. On the other hand, 12.5% of positive words were produced by the moderate group, compared to 10.22% for the severe group. Six overall categories were identified, and each was split into two subcategories according to their positive or negative valence. The six categories were: good or poor evaluation of the body, good or poor level of energy, relaxed or tense body, dys/functional, positive/ negative physical image, physical pain or not (Figure 2).

Table 2 provides an example of a positive and negative word for each category.

### Post-program Description of the Population

The average age of the 26 volunteers who agreed to participate in the post-program sessions was 34.3 (SD: 6.51), and the mean PCL5 score was 58.56 (14.45). Pre-session, a moderate positive correlation was observed between the FMI-A score and RMSSD (*r*^2^ = 0.39; *p* = 0.09) in this group.

Following the program, the mean PCL5 score was 52.8 (14.54). A significant decrease was observed (Z = 2.56; *p* = 0.01). However, in only one case did the post-session score fall under the cut-off of 33. The decrease in the PCL5 score was associated with an increase in the mean overall FMI score (Z = 3.07, *p* = 0.003), the mean FMI-P score (Z = 1.79, *p* = 0.074), and the mean FMI-A score (Z = 3.2, *p* = 0.002). A decrease in RMSSD was also observed (Z = 3.09; *p* = 0.002). Strong positive correlations were observed pre- and post-session for the PCL5 score, the overall FMI score, and its sub-scores (0.60 < *r*^2^ < 0.90; 0.0001 < *p* < 0.002). No correlation was observed pre- and post-session for RMSSD. At post-session, a moderate positive correlation was observed between the overall FMI score and RMSSD (*r*^2^ = 0.41, *p* = 0.08).

Pre-session, participants gave 127 words to describe themselves and their body; of these, 37.8% were positive, and 62.2% were negative. Post-session, 111 words were given; none of these were considered neutral, 59.45% were positive, and 40.54% were negative. The analysis of the valence of these words found an increase in the total number of positive words (both referring to the person and their body) between pre- and post-session (*X*^2^ = 10.29; *p* = 0.001).

**Figure 3:**
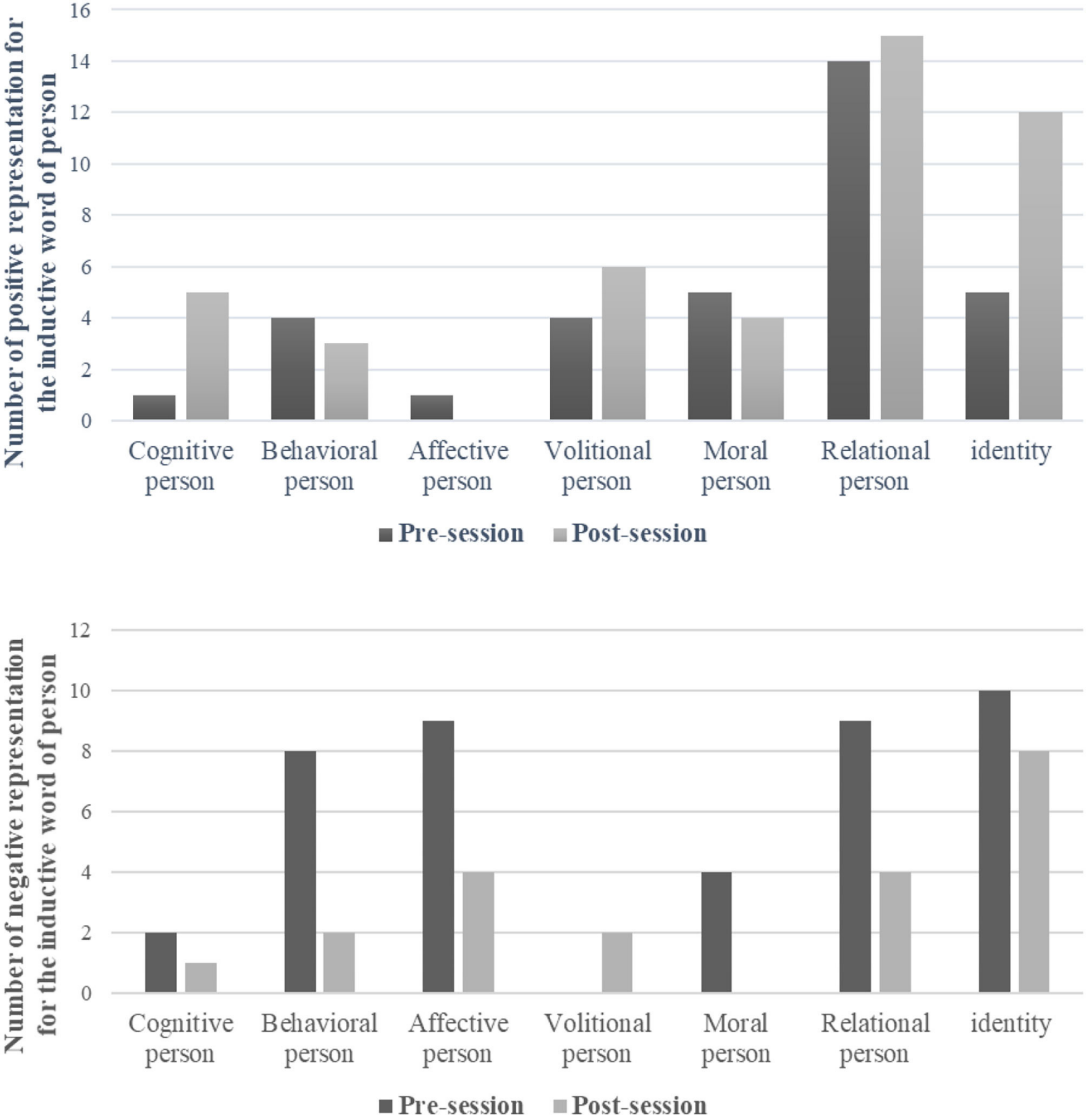
Categories and numbers of positive (top) and negative (bottom) words used to refer to the person at pre-session and post-session.

Pre-session, participants gave 76 words to describe themselves; of these 46% were positive, and 54% were negative. Post-session, 66 words were given; 68% were positive, and 32% were negative. The analysis found a significant increase in the number of words with positive valence (*X*^2^ = 6.16; *p* = 0.01). Figure 3 shows the number of words referring to the person as a function of the seven categories, pre- and post-session. It should be noted that the category of “moral” disappeared.

**Figure 4:**
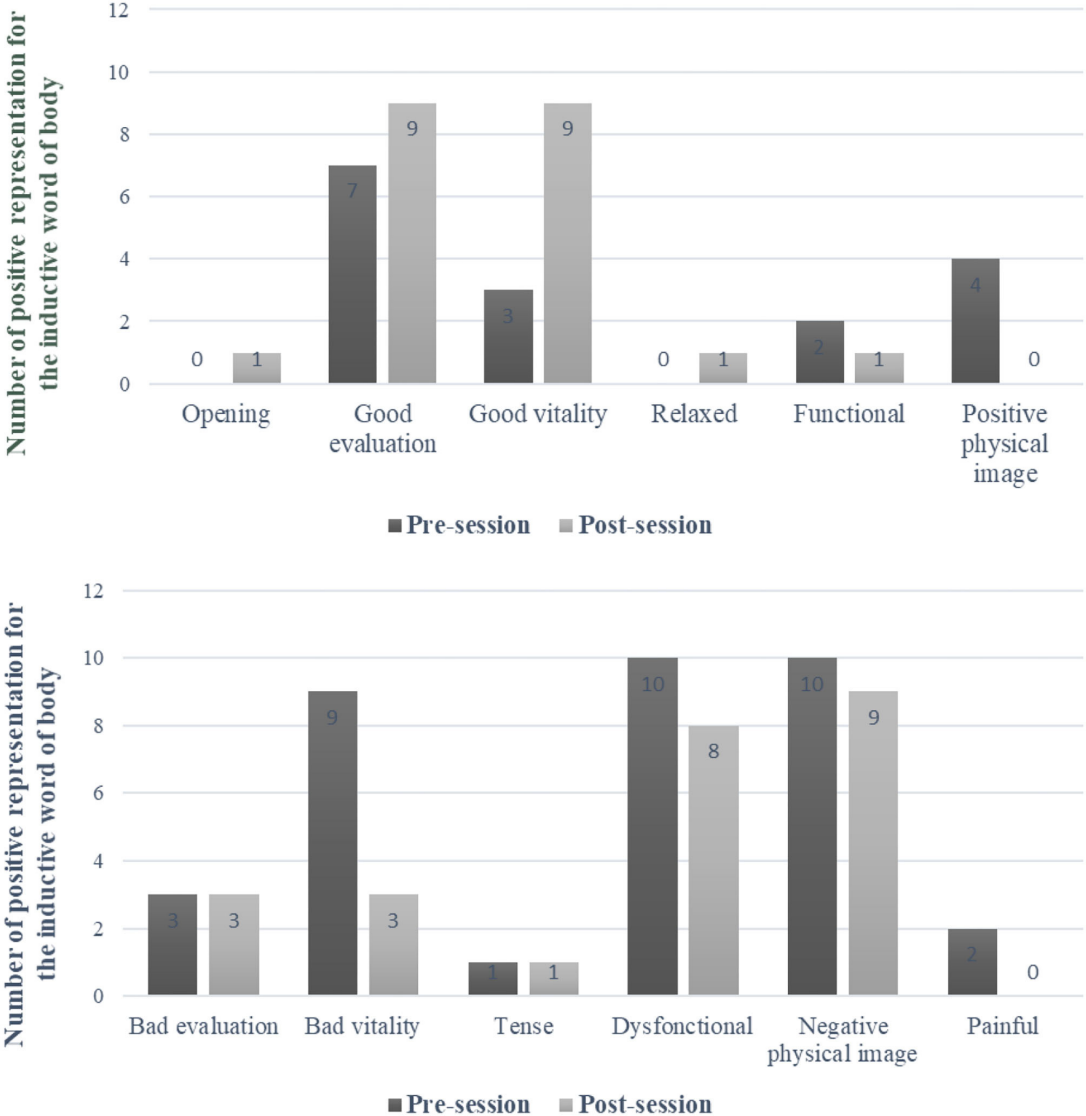
Categories and numbers of positive (top) and negative (bottom) words used to refer to the body at pre-session and post-session.

Turning to representations of the body, 51 words were given pre-session; of these, 25% were positive, and 75% were negative. Post-session, 45 words were given; of these, 47% were positive, and 53% were negative. The analysis identified evidence of a trend with respect to an increase in the number of words with positive valence (*X*^2^ = 3.8, *p* = 0.05). Figure 4 shows the number of words used to describe the body, pre- and post-session, according to the categories listed above. Three changes were observed: (i) the painful category disappeared; (ii) a positive word appeared in the relaxed/ tense category; and (iii) a new opening/ closing category emerged, with one positive word used to describe the body.

## Discussion

This study examined a population of military veterans with chronic PTSD, who had been on sick leave for more than 6 months.

The overall analysis of verbal descriptions of the self-found several differences with respect to the valence and semantic characteristics of words used to refer to the body and the person. The identified categories are part of the cognitive-behavioral theoretical approaches. There is no single definition of cognitive-behavioral theory because there are so many different cognitive-behavioral theories. However, all cognitive-behavioral theorists value the role that cognitions play in the development and maintenance of psychological problems (47). Targeted categories, namely semantic categories, may offer therapeutic approaches to the management of PTSD in veterans. In absolute terms, the biggest category was negative representations of the person; this was followed by negative representations of the body, positive representations of the self, and finally, positive representations of the body. On a semantic level, descriptions of the person could be divided into seven categories: cognitive, behavioral, affective, volitional, moral, relational and identity. Most positive representations fell into the relational category, while only one positive word was classified as affective. Negative descriptions were distributed across all seven categories, but behavioral, affective, relational and identity were particularly important.

Representations of the body were divided into six categories: good or bad evaluation, good or bad vitality, relaxed/tense, dys/functional, physical image, no/pain. The “relaxed/tense” category was the only one that did not contain a positive word (indicating a relaxed state). These representations provided an insight into SRP among veterans.

Our overall population was divided into two groups consisting of those with either severe or moderate PTSD. Between-group comparisons were carried out at the vagal level, and with respect to the mind–body connection, as both are known to be related to interoception. The analysis showed that those with moderate PTSD tended to have a higher level of parasympathetic functioning, but that this was not associated with differences in terms of the mind–body connection. Further comparisons of representations of the self-showed that, contrary to our hypothesis, those in the severe group did not produce more negative words than those in the moderate group. We conclude that the self-representation appears to be independent of PTSD severity for veterans who have suffered from PTSD for at least 6 months. With regard to interoception, no connection could be identified between the introspective evaluation of the self, and the psychophysiological evaluation of interoception.

For subjects who participated in the post-program session, the analysis found that participation in the course was associated with a decrease in clinical PTSD severity, and an increase in the mind–body connection, notably the acceptance dimension. Although RMSSD decreased post-session, we found no relationship with the pre-session level. However, a moderate positive correlation was found between post-session RMSSD and the mind–body connection. Thus, we conclude that the main effects of the sports program related to subjective evaluations of PTSD severity, and mind–body functioning. However, our results are too tenuous to draw any conclusions in terms of interoception improvement.

In accordance with our second hypothesis, the benefits of the program were associated with a positive change in the self-representation. First, we observed a general increase in positive representations, for both the person and their body. While both increased, the increase was higher for the person than the body. Second, the categories changed. For person representations, the category of “negative moral” disappeared. For body representations, the “painful” category disappeared and a positive word appeared in the “relaxed/tense” category. Furthermore, the opening/closing body category emerged, and one word was classified as positive (opening).

These positive changes appeared at the end of the 9-day sports program, and suggest that these veterans may have reconnected with aspects of their previous identity and sense of self. They also appear to have a more positive relationship with their body, suffer less pain, be more relaxed, and be more dynamic. However, here again, the introspective evaluation of the self through the analysis of words could not be related to an improvement in variables related to interoception, especially as post-program, parasympathetic activity decreased. This decrease in RMSSD at the end of the program appears to be inconsistent with the observed clinical improvement, the better body–mind connection, and the emergence of a more positive self-representation.

HRV is a widely-accepted indicator of psychological stress (48). However, stress is known to be consistent with a wide range of individual differences in the autonomic response—from sympathetic activation to vagal withdrawal, or a reciprocal pattern of autonomic response (48). On the one hand, the observed post-session decrease in parasympathetic tone at rest suggests that the program as a whole, including the workshops, sports activities and life outside the home, disrupt homeostatic processes (49). Each activity is a challenge for the veterans. Participants describe themselves as very tired at the end, especially as many have resumed physical activity after a long pause during their sick leave. On the other hand, the concomitant improvement in clinical symptoms, and the mind–body connection, suggests that the program may constitute a positive challenge that contributes to the recovery of the self. Only a further long-term assessment can answer this question. Finally, the rest condition used in the vagal assessment is currently recognized as insufficient. There is a need to assess the three systematic levels of cardiac vagal control (resting, reactivity, and recovery) in order to fully evaluate how efficiently self-regulatory resources are mobilized and used (50).

This exploratory study has several limitations. The first is the population, which is only composed of male veterans. This implies that any generalization to other populations must be undertaken with caution. Second, the small number of subjects means that the analysis lacks statistical power. Third, several limitations relate to the evaluation of interoception. In this study, we indirectly assessed interoceptive sensitivity, which refers to the degree to which the individual feels engaged by interoceptive signals, with a mind–body connection scale. Our subjective measure of interoceptive sensitivity needs to be supplemented by a behavioral task in order to complete the assessment. This is all the more important given that subjective sensitivity to interoceptive signals does not account for cerebral activation. The latter observation makes any interpretation of the relationship between the benefits of the program for interoception, and improvements in the self-representation, questionable. Turning to the physiological interoception assessment, RMSSD was only recorded at rest, and this measure should be supplemented with measures of vagal reactivity and recovery. Finally, three limitations come from the protocol. The effects of the 9-day program on self-representations and symptomatology are not controlled. The program included both workshops and sports, the observed benefits could be attributed to the workshops, the sporting activities, or both. Self-selection bias could be due to the post-program sample. These three methodological limitations make any generalization of the results questionable.

## Conclusion

This exploratory study aimed to provide an insight into how military veterans evaluate their self. Most representations of the self, notably the person and their body, are negative, illustrated by the violence of some of the words used by the cohort of veterans. At the same time, the negativity of these words was found to be unrelated to clinical PTSD severity. Sport could be a way to reappropriate a positive image of the self. The findings of this study suggest that sport could be used to improve the individual's representation of the person her or himself, by improving the representation of the body. Although this relationship could be consistent with better interoception, our results are inconclusive on this point.

## Data Availability Statement

The raw data supporting the conclusions of this article will be made available by the authors, without undue reservation.

## Ethics Statement

This study received the agreement from the Sud-Mediterranée III personal protection committee before the start of inclusions, 10 of September 2018 (NTC03995992). All the subjects received information on the protocol and gave their written consent prior to their participation. The patients/participants provided their written informed consent to participate in this study.

## Author Contributions

MT designed the study. AB, AD, CM-K, DL, GL, SJ, and MT planned and participated in word and statistical analyses. CB, GL, AB, SJ, and MT recorded the data. CB, CM-K, DL, and MT participated in all stages of writing and proofreading. All authors took an active part in the process and read and approved the final manuscript.

## Funding

The work was supported by a grant (80,000 euros) from the Gueules Cassées.

## Conflict of Interest

The authors declare that the research was conducted in the absence of any commercial or financial relationships that could be construed as a potential conflict of interest.

## Publisher's Note

All claims expressed in this article are solely those of the authors and do not necessarily represent those of their affiliated organizations, or those of the publisher, the editors and the reviewers. Any product that may be evaluated in this article, or claim that may be made by its manufacturer, is not guaranteed or endorsed by the publisher.
